# Editorial: An insight into university medical and health science courses

**DOI:** 10.3389/fpubh.2022.1074966

**Published:** 2022-11-22

**Authors:** Sunjoo Kang, Melody S. Goodman, Harshad P. Thakur, Michal Grivna, Sanjay P. Zodpey

**Affiliations:** ^1^Department of Global Health, Graduate School of Public Health, Yonsei University, Seoul, South Korea; ^2^Department of Biostatistics, School of Global Public Health, New York University, New York, NY, United States; ^3^Centre for Public Health, School of Health Systems Studies, Tata Institute of Social Sciences, Mumbai, India; ^4^Institute of Public Health, College of Medicine and Health Sciences, United Arab Emirates University, Al Ain, United Arab Emirates; ^5^Public Health Foundation of India, New Delhi, India

**Keywords:** insight into university, medical, health science, online education, equity

## Introduction

As emerging infectious diseases (EID) are expected to occur, healthcare personnel must prepare medical and health science majors on the effects of public health crises and the epidemiology of EID. The courses designed to increase interdisciplinary research competence are being developed by higher education institutions with the goal of reducing the disparities in education based on the level of socioeconomic development among countries (1). A greater understanding of environmental health determinants is emphasized in medicine (2), and crisis leadership has been required in public health education over the last 2 years of the coronavirus disease 2019 (COVID-19) pandemic (3).

However, optimistic interpretations of the mandatory application of online education as replacements for offline courses under COVID-19 exist. While online education is accessible from anywhere, it also has several limitations. Instructors lacked technological skills, such as experience with ZOOM or TEAMS, video-recording of presentations, educational experience with online education, how to keep the attention of online students, and so on. Environmental infrastructure and properly trained instructors are necessary to ensure effective, high-quality online education (4). Opportunities such as Internet access, educational material download, and two-way communication are assumed to be provided equally so that learners can share their experiences and ideas even with time barriers (5, 6). However, the effect of online education experienced by learners differs according to the national income level, infrastructure of the Internet, and information communication technology. To supplement practice education, it is necessary to use a video display terminal in augmented reality technology or search for other alternatives (7). Unlike advanced countries such as the UK and US, which applied online education more than 10 years before the COVID-19 pandemic, most educational institutions and learners in developing countries such as Iran, Uganda, and Pakistan negatively evaluated online learning experiences and effects (5, 8). However, medical students in Belgium responded that the quality of education decreased, but self-directed learning competence developed during the pandemic (9, 10).

Fourteen articles were selected for publication. Most research findings have addressed medicine or public health curriculum and educational content improvement, the performance of public health degree programs, education to strengthen research capabilities, health education for specific diseases and subjects, and simulation education or augmented reality; however, only one study has examined the effect of online education during the COVID-19 pandemic. Five articles address pre-service education issues (Zhang et al.; Sun et al.; Syed et al.; Tu et al.; Yang et al.). One addresses in-service education issues (Yang et al.). Six articles are geared toward holistic or programmatic aspects (Zhang et al.; Rao et al.; Li et al.; Shete et al.; Dinov; Anitha et al.) with two disease or module-specific articles (Liu et al.; Jia et al.).

For this Research Topic, we received various types of articles, namely, original research, perspectives, brief research reports, mini-reviews, systematic reviews, curricula, instructions, and pedagogies. The authors' works are summarized according to the article type.

## Contributions according to article type

### Perspectives

As the public health crisis continues, the impact of environmental factors on health and the competencies of crisis management is reinforced to respond effectively to emerging infectious diseases in the future. Rao et al. argued that an understanding of the public health context and the impact of socio-ecological factors on health in medical education was necessitated by all types of undergraduate and special short-term courses in the United States. These expectations in medical education peaked throughout the COVID-19 pandemic because the role of clinical doctors was expanded to individual disease treatment and expert competencies in response to emerging infectious diseases. Recommendations for the reform of medical education were made on seven topics: premedical education, Medical College Admission Test, public health and nutrition course within the first 2 years of medical school, post-coursework or standardized licensure testing, research within training, clinical practice, and continuing education.

### Original research

Healthcare personnel is essential to achieve sustainable development goals by 2030 and respond to the demand for health services during the public health crisis; therefore, higher education in medicine and health science should include the following: innovation in the course content, application of advanced and effective teaching and learning methods, quality management of the clinical practice, performance evaluation of programs, responsiveness to the provision of health services, and appropriate health education for communities.

Tu et al. conducted an online survey of 8,285 students of college level and higher from Jiangsu province, China, to identify their current knowledge, attitude, and sex education on Human Immunodeficiency Virus/Acquired Immunodeficiency Syndrome (HIV/AIDS). The authors determined the influencing factors of HIV/AIDS knowledge and students' health needs to be based on their knowledge using the structural equation model. Their findings indicate that HIV/AIDS health education has a significant effect on knowledge awareness and attitude, with standardized coefficients of 0.15 and 0.58, respectively. The authors concluded that timely sex education should be implemented to decrease the incidence rate of HIV/AIDS among young people. Syed et al. at King Saud University in Saudi Arabia examined undergraduate health science students' knowledge and perceptions of thyroid cancer development. The findings of their cross-sectional survey showed that female students had a significantly higher level of thyroid cancer knowledge and perception of the causes of the disease than male students (*p* < 0.05). More than half of the students (65%) were well aware of unexplained lumps or swelling as a warning sign of thyroid cancer. The authors concluded that the findings shed light on the understanding of pharmacology, nursing, and medical students' knowledge of thyroid diseases and highlighted the need for further repetitive qualitative studies that include participants from various institutions.

The COVID-19 pandemic played a role in advancing digital transformation by unexpectedly applying online education to university education, which had to prepare for innovative teaching and learning methods during the Fourth Industrial Revolution. In this regard, Sun et al. conducted a descriptive study using massive open online courses (MOOCs) platform data of course-taking students and official socioeconomic statistics in China. The relationship between socioeconomic factors and the number of participants during the COVID-19 pandemic was analyzed. The authors found a close relationship between the number of MOOCs course-takers and regions of China only during the COVID-19 pandemic. Their findings are important in understanding the inequality of access to MOOCs and the disparity of educational opportunities according to regional and socioeconomic status (i.e., the digital divide).

Simulation is applied at the level of modality in healthcare for education, assessment, research, and facilitating patient safety. As hospital-based clinical training has been restricted to prevent social distancing and infection after the COVID-19 outbreak, simulation education to replace practice training in the simulation lab of nursing and medical schools has drawn attention. Zhang et al. identified the characteristics of virtual simulation (VS) and examined the trends in nursing research. The authors reviewed 677 articles from 1999 to 2021 with identifiers of authors, institutions, countries, journals, and network maps using bibliometric analysis. Their findings showed that the number of studies peaked exponentially in 2020 by authors from the US, Canada, and Australia. These studies were published mostly in three journals: *Clinical Simulation in Nursing, Nursing Education Today*, and the *Journal of Nursing Education*. The authors divided the four major themes into the following clusters: (i) virtual learning during the COVID-19 pandemic as Cluster 1, (ii) clinical nursing care using virtual reality as Cluster 2, (iii) education in nurse practitioners using vs. as Cluster 3, and (iv) education technology in vs. as Cluster 4. They concluded that further programs are required on training and evaluation outcomes using vs. for nursing students and faculty training on the use of VS.

Yang et al. examined the effects of a novel simulation curriculum on pediatric cardiovascular education for residents at a children's hospital in Hangzhou, China. The non-equivalent control group pre-and post-test design with the three segments of running, debriefing, and challenging task provision was applied only to the intervention group. The authors found that the assessment results of residents' knowledge (*p* < 0.01), skills (*p* < 0.01), professionalism (*p* < 0.05), and self-performance satisfaction feedback (*p* < 0.05) significantly increased in the intervention group only after simulation training. The authors concluded that qualified simulation training should be provided to the residents to enhance professionalism and pediatric cardiovascular knowledge and skills.

The mission of higher education institutions is to educate and produce quality health and medical personnel, as required by modern society. To pursue this goal, university education institutions should periodically identify and analyze changes in the health and medical environment to continuously improve the performance of educational programs. In this regard, Shete et al. conducted research on the outcomes of a university's training program in implementation science. For the implementation sciences, the training program of the University of California, San Francisco, revised domains into seven categories: team science, context identification, literature identification and assessment, community engagement, intervention design, and research implementation, evaluation of the effect of translational activity, and behavioral change communication strategies. The survey and academic productivity of the program participants in both in-person and online programs were assessed. The findings of this study showed that participants from both training programs had high to moderate confidence in all 12 competencies, with 181 publications. Only one competence in intervention evaluation was low because of the early stage in the career ladder. The authors concluded that a qualitative assessment with a larger sample size should be considered in future studies.

To identify graduates' distribution in the health employment industry, Li et al. examined the occupational situation of master's graduates in public health and preventive medicine in China. A cross-sectional survey was conducted to collect data from 2014 to 2018 (i.e., 5 years of program graduates). The findings of the study showed that most participants (95%) were employed in hospitals, and the jobs were not matched with their major and had a low initial salary. The rate of job maintenance was as high as 83%. The authors concluded that initial salary and job arrangement should be matched to their specialty by remodeling the programs and advocating for changes in employment policy.

In health sciences and medical education, graduates' proactive lead in research performance ability is considered an important achievement. Yang et al. conducted a study on the effectiveness of student-driven course-based undergraduate research experiences (CUREs) at a medical school in Shanghai, China. The authors identified the experience of general bacterial culture and gene amplicon sequencing under the guidance of instructors at the Shanghai Jiao Tong University School of Medicine. Their findings showed that CUREs contributed to the improvement of students' hard skills (research performance processes based on scientific theory, etc.) and soft skills (design, performing research, result in production, etc.). The authors concluded that CUREs were beneficial to scientific literacy skills and understanding instructors' roles from the student's perspective.

Intake of chronic disease medications among older adults in the community is an important issue in managing their healthy living without complications or aggravation of the disease. Considering one-third of older adults' medication non-adherence in Beijing, China, Jia et al. examined the relationship between health literacy and medication adherence among community older adults in Beijing, China, using a cross-sectional survey. The authors analyzed the association between the cognitive level of participants and sociodemographic factors on medication compliance. Their findings revealed that the lower the literacy rate, the higher the non-compliance with drug use (*p* 0.01); however, no relationship was observed between the two variables in the case of cognitive impairment. The authors concluded that medication adherence should be monitored with older adults' literacy ability after assessment of the cognitive level classified into normal and impairment levels.

### Mini-review

Globally, the public health curriculum—as an undergraduate or master's degree program—has expanded through the approval of programs or certified curriculum systems in advanced countries since 1990. Given the importance of public health education and its competencies, Anitha et al. analyzed 180 public health degree programs in seven South Asian countries (India, Bangladesh, Pakistan, Bhutan, Maldives, Afghanistan, and Sri Lanka), and they formed the South Asian Association for Regional Cooperation. Most countries, except India, still do not operate an educational accreditation or licensure system or regulations for public health education programs. Public health education in India is governed by a new national education policy that provides more research-based learning. However, public health professionals can play major roles in primary healthcare for universal health coverage and health literacy for communities in resource-scarce underdeveloped countries. Suggestions for the improvement of the enrollment of public health majors students and the accreditation system to evaluate the programs will contribute to the health workforce industry and the quality of public health programs in South Asia.

### Brief research report

Meta-verse, the main technology of the fourth Industrial Revolution, is a three-dimensional virtual world, and the market has grown rapidly owing to the popularization of its use in realistic content.

The effectiveness of education has been proven through the meta-verse platform, augmented reality, and virtual reality education cases and the current use of video display terminals (VDT) is expected to continue to increase in clinical training for healthcare personnel and undergraduate simulation practicum.

Liu et al. analyzed the relationship between the total duration of daily VDT use, and the level of eye discomfort among medical students in a cross-sectional study in Kunming, Yunnan province of China. The study findings showed that the prevalence rate of VDT in medical students was significantly higher than that reported in 2016; however, the result may be affected by the COVID-19 pandemic and disruption of on-site clinical practice. A statistically significant positive relationship was observed between the total duration of VDT use and the severity of ocular discomfort among the participants (*p* < 0.05). The severity of eye discomfort symptoms was negatively affected by the total sleep duration and positively affected by the total VDT use duration (*p* < 0.05).

### Curriculum, instruction, and pedagogy

Big biomedical data (as an enabler for a knowledge society) and data analytics (as an asset platform) are mandatory for health science professionals during the Fourth Industrial Revolution and digital transformation. Dinov reviewed advanced teaching and learning methods and the content of courses focused on the competence of analytics for doctoral programs in health science. Any doctoral program on health science majors, biomedical, informatics, and health analytics training should incessantly include the demand for big data literacy and analytics in their advanced curriculum framework. To meet these expectations, various courses on data quality, model interpretability with transparency, research ethics, information security and privacy protection, health policy overviews, and quantitative analysis should be incorporated. Recommendations on the areas of expected program graduates' competencies include algorithms and applications, data management, and analysis methods. The perspective of transdisciplinary efforts considers innovation-taking risk and uncertainty, but it is better than being ostracized by complacency.

### Systematic review

The teaching and learning methods for nursing, medical, and public health majors were designed to provide more realistic practice opportunities because of patients' right to self-determinism and protection of patient privacy. Clear learner expectations of their teaching behaviors would be beneficial to clinical instructors. In this regard, Zhang et al. meta-reviewed nine articles based on the Joanna Briggs Institute Qualitative Assessment and Review Instrument (JBI-QARI). The three themes of good teaching literacy, solid professional competence, and harmonious relationships were synthesized using the JBI-QARI after category grouping. The good teaching literacy theme was synthesized from the categories of flexible teaching methods, a mature concept of education, and good personality characteristics. Solid professional competence was synthesized from the categories of excellent theoretical knowledge and operational skills, good professional attitude, and rich teaching experience. The harmonious faculty-student relationship was synthesized from the categories of the relationship of trust and objective evaluation. The authors concluded that qualitative analysis provided a better understanding of their relationship. They focused on awareness of nursing students' expectations during clinical practice to ensure effective students experience by clinical nursing teachers.

## Conclusion

The studies highlighted herein provide insight into university medical and health science courses and have diverse contributions. Perspectives have been offered on training frontline healthcare personnel to fulfill the societal mandate in the era of the Fourth Industrial Revolution.

Original research reiterates the importance of university course reform in understanding students' outcomes and details how to achieve program performance through quality instructors; it also highlights the benefits of applying the advanced method of simulation and evidence-based research competence.

## Author contributions

SK conducted the literature review and prepared the draft of Editorial in discussion with MG and HT. This work was carried out in collaboration with all authors. All authors read, revised, and approved the final manuscript.

## Conflict of interest

The authors declare that the research was conducted in the absence of any commercial or financial relationships that could be construed as a potential conflict of interest.

## Publisher's note

All claims expressed in this article are solely those of the authors and do not necessarily represent those of their affiliated organizations, or those of the publisher, the editors and the reviewers. Any product that may be evaluated in this article, or claim that may be made by its manufacturer, is not guaranteed or endorsed by the publisher.
